# Development of Invisible Sensors and a Machine-Learning-Based Recognition System Used for Early Prediction of Discontinuous Bed-Leaving Behavior Patterns †

**DOI:** 10.3390/s20051415

**Published:** 2020-03-05

**Authors:** Hirokazu Madokoro, Kazuhisa Nakasho, Nobuhiro Shimoi, Hanwool Woo, Kazuhito Sato

**Affiliations:** 1 Faculty of Systems Science and Technology, Akita Prefectural University, Yurihonjo City, Akita 015-0055, Japan; shimoi@akita-pu.ac.jp (N.S.); woo@akita-pu.ac.jp (H.W.); ksato@akita-pu.ac.jp (K.S.); 2 Faculty of Engineering, Yamaguchi University, Ube City, Yamaguchi 755-8611, Japan; nakasho@yamaguchi-u.ac.jp

**Keywords:** ambient sensors, home agent, life monitoring, machine learning, quality of life, random forest

## Abstract

This paper presents a novel bed-leaving sensor system for real-time recognition of bed-leaving behavior patterns. The proposed system comprises five pad sensors installed on a bed, a rail sensor inserted in a safety rail, and a behavior pattern recognizer based on machine learning. The linear characteristic between loads and output was obtained from a load test to evaluate sensor output characteristics. Moreover, the output values change linearly concomitantly with speed to attain the sensor with the equivalent load. We obtained benchmark datasets of continuous and discontinuous behavior patterns from ten subjects. Recognition targets using our sensor prototype and their monitoring system comprise five behavior patterns: sleeping, longitudinal sitting, lateral sitting, terminal sitting, and leaving the bed. We compared machine learning algorithms of five types to recognize five behavior patterns. The experimentally obtained results revealed that the proposed sensor system improved recognition accuracy for both datasets. Moreover, we achieved improved recognition accuracy after integration of learning datasets as a general discriminator.

## 1. Introduction

Aging in Japan has been progressing rapidly not only because of an increasing number of elderly people and their longevity, but also a decreasing number of young people caused by a declining birthrate. Although the demand for nursing-care services has been growing along with the continuously developing aging society, the supply is insufficient because of changing demographics [1]. As an occupational characteristic of caregivers, the occupation and turnover rates are both high compared to those of other industries [2]. Caregivers must work not only to provide various care services physically and mentally, but also for shifting during nighttime for providing 24-hour nursing services and support. Especially for nighttime, a severe caregiver shortage leads to insufficient nursing-care, which involves a risk of inducing accidents related to the daily life for care recipients.

Mitadera et al. [3] reported that fall accidents of elderly people accounted for more than 50% of all accidents at nursing-care facilities. Situational details reveal that most accidents occurred when elderly people left their bed and its surroundings. Moreover, 85.5% of fall-related accidents occurred under circumstances without assistance or supervision. Therefore, preventive measures using bed-leaving sensors are indispensable for detecting bed-leaving behavior in an early stage because facility administrators are charged with management responsibility if an accident occurs.

Recently, various bed-leaving sensors are commercially available from manufacturers. For example, clip sensors, mat sensors, infrared (IR) sensors are widely used at hospitals and nursing-care facilities. Although clip sensors are used easily because they are the most reasonable means available, care recipients are restrained by sensor wires because they are attached directly to the patient’s nightwear. Moreover, a risk exists that a sensor wire might wrap around the neck of a care recipient. Therefore, the use of clip sensors has been discouraged recently. Mat sensors, which are inexpensive even compared with clip sensors, are used widely at clinical sites because they entail no restraint. One shortcoming of mat sensors is their slow detection and response from the position where a care recipient sits at the bed terminal while putting their feet on it. Another shortcoming is the excessive reaction even when a caregiver or a family member passes through while stepping on it. Furthermore, a care recipient might attempt to leave while consciously avoiding stepping on a mat sensor because it is a visible sensor. IR sensors present similar shortcomings to those of mat sensors. Moreover, caregivers must check the sensor installation status because care recipients touch them occasionally.

To prevent fall accidents, sensor systems with bed-leaving behavior predictions at an early stage have been studied. Asano et al. [4] proposed a detection system using a depth camera. They employed support vector machines (SVMs) to recognize bed-leaving behavior patterns after optimizing parameters and motion features combined with the body size, position, and orientation of respective subjects. Their experimentally obtained result achieved 92.65% recall after 68 iterations. However, the precision was insufficient for practical application because false detection occurred in within 24 iterations. Moreover, they used a depth camera for capturing images. Although it is difficult to identify profiles solely from depth images, a challenging task remains: eliminating unpleasantness felt by a patient who is monitored by a camera.

Kawamura et al. [5] proposed a wearable sensor system using a three-axis accelerometer. For their system, unrestrained measurements are actualized using a lightweight sensor module of 13 g. With consideration of clinical applications, they obtained not only metaparameters that contributed to recognition, but also experimentally obtained results with bed-up and wheelchair locomotion. No recognition accuracy was provided as detailed sensor characteristics. Moreover, they provided neither recognition accuracy nor detailed sensor characteristics. Existing bed-leaving sensors entail persistent difficulties related to quality of life (QoL), detection speed, convenience, and cost. No sensors that satisfy these requirements have been put to practical use. In modern society, the declining birthrate and aging population are progressing rapidly. We therefore regard development of sensor systems that overcome these problems as an urgent task to.

This study was conducted to develop a bed-leaving recognition sensor system that is inexpensive, convenient, and maintainable with advanced QoL for care recipients. For improving recognition accuracy and reliability compared with our earlier bed-leaving sensor system [6], our novel sensor prototype comprises pad sensors and a rail sensor installed respectively on a bed frame and a bed-side safety rail. For evaluating our sensor system, we obtained original benchmark datasets of two types: continuous datasets with behavior transitions from sleeping to bed leaving in a predefined interval and discontinuous datasets with free and random movements obtained from 10 subjects. We compared machine learning algorithms of five types to recognize five behavior patterns. Our earlier study [6] provided respective classifiers because bed-leaving behavior patterns have unique characteristics along with their subjects. Nevertheless, for this learning strategy, the recognition accuracy was insufficient to ensure reliability for discontinuous datasets. Therefore, we developed a single classifier using all continuous datasets. The experimentally obtained results revealed that the proposed sensor system improved recognition accuracy for both datasets.

The rest of the paper is structured as follows. In Section 2, our originally developed sensors of two types and their measurement system are presented. Section 3 and Section 4 present our proposed method based on machine learning algorithms of five types and our original datasets obtained from ten subjects, respectively. Subsequently, Section 5 presents evaluation results with recognition accuracies and confusion matrixes in respective datasets, respectively. Finally, Section 6 concludes and highlights future work. Herein, we had proposed this basic method with originally developed sensors of two types in the proceeding [7]. Moreover, we had presented basic characteristics of pad sensors in the proceeding [8]. For this paper, we have described detail results and discussion in Section 5.

## 2. Sensor System

### 2.1. System Structure

**Figure 1:**
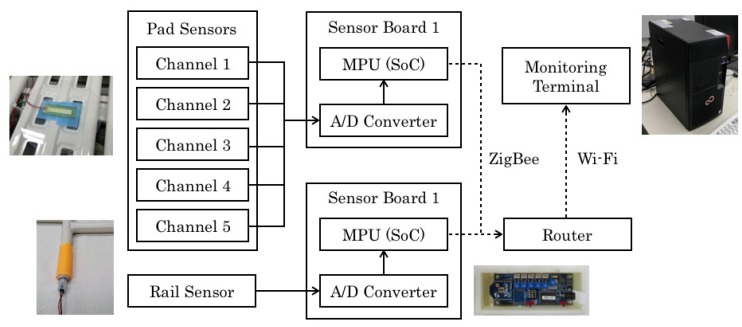
Whole system structure.

Figure 1 depicts the whole system structure of our novel sensor prototype, which comprises pad sensors, a rail sensor, sensor boards, a wireless router, and a monitoring computer. Output signals are collected to the sensor boards with a wired connection. The sensor boards convert analog signals to digital signals in real time. Digital signals are sent to a monitoring computer with wireless connection. We used ZigBee, a short distance wireless communication protocol, to provide cost-effective implementation and low power consumption. The transmitted measurement signals are displayed on the monitoring computer in real time. Behavior recognition algorithms based on machine learning are incorporated in the monitoring computer.

### 2.2. Pad Sensor

**Figure 2:**
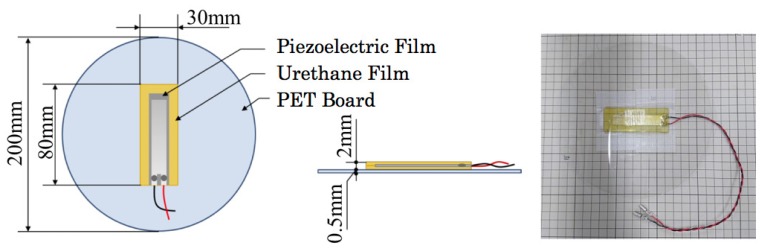
Interior architecture and pad sensor appearance.

Figure 2 depicts our originally developed pad sensor prototype. The pad sensors are non-restrictive, invisible, cost effective, and independent of sensor driven power. We used a piezoelectric film, which generates a potential difference when distortion occurs in an arbitrary direction against external forces such as receiving vibration. A commercial piezoelectric film (DT2-028K/L; Tokyo Sensor Co., Ltd.) is sandwiched by 1-mm-thick urethane sheets with 50 deg hardness. The durability and elasticity of the piezoelectric film are improved using urethane sheets. Moreover, polyethylene terephthalate (PET) boards with a larger size than the urethane sheets provide extended sensing ranges. The sensor core is protected using PET plates that have 200 mm diameter and 0.5 mm thickness. We used ultraviolet stiffened resin to adhere to the piezoelectric film and the urethane sheets.

### 2.3. Rail Sensor

Care recipients whose legs and body are weak sometimes grip a safety rail beside a bed when they try to stand up. Motegi et al. [9] reported that approximately 82% of care recipients gripped a safety rail when they left from their bed. Therefore, we specifically examined a bed side safety rail for the prevention of a fall. We consider that the recognition accuracy is improved if gripping of a safety rail is detected using a dedicated sensor.

**Figure 3:**
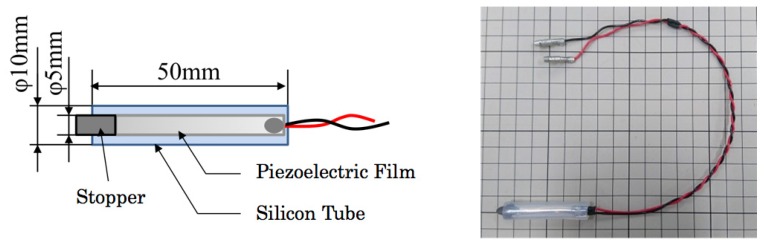
Interior architecture and appearance of the rail sensor.

Figure 3 depicts our originally developed rail sensor prototype. We inserted a piezoelectric film, DT2-028K/L, into a silicon tube with 50 mm length, 10 mm outer diameter, and 5 mm inner diameter. As a stopper and a protector, a metal cap is stuffed to the sensor upside.

### 2.4. Basic Characteristics

**Figure 4:**
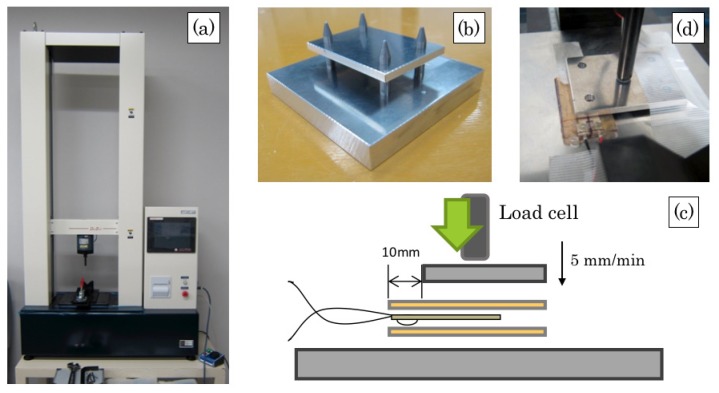
Load test: (**a**) fixture, (**b**) schematic diagram, (**c**) load test, and (**d**) the load test machine (Multi Force Analyzer FWT-1000; DigiTech Co. Ltd., Osaka city, Japan).

For evaluating characteristics of our developed film-load sensors, we conducted preliminary experiments using the load test machine (Multi Force Analyzer FWT-1000; DigiTech Co. Ltd.), as depicted in Figure 4a. The major specifications of the machine are: 1 kN rated weight; 100 mN resolution; 600 mm/min maximum test speed; and ±0.2% weight precision. The majority of loads are attained from the vertical side as a surface load because of the installation of the sensors on the bed frames. For this load test, we developed a fixture made of A2017 duralumin, as depicted in Figure 4b. The major specifications of the fixture are 100 × 100 mm with 15 mm basement thickness and 70 × 50 mm with 5 mm top thickness.

The load reaches the maximum for longitudinal sitting, which raises the upper body of a person to start leaving the bed. From the report [10] of human sciences of nursing, the body weight to the hip at longitudinal sitting is approximately equal to the total weight of the upper body. It is 87% of the total body weight. According to the National Healthcare and Nutrition Report 2014 in Japan [11], the mean weights of people older than 65 years old people are 61.9 kg for men and 50.8 kg for women. Based on both mean weights, we set test loads from 340 N to 680 N for five sampling points.

We evaluated the output characteristics of our developed sensors of five sets with the default test speed of 5 mm/min. Figure 4c depicts a schematic diagram of the sensor output that occurs from the range except that of the rivet parts. For attaining a load, the sensor is fixed to the removal part of 10 mm from the boundaries. We measured output voltages from respective sensors using a data logger (LR8431; Hioki Corp.) concomitantly with the test load.

**Figure 5:**
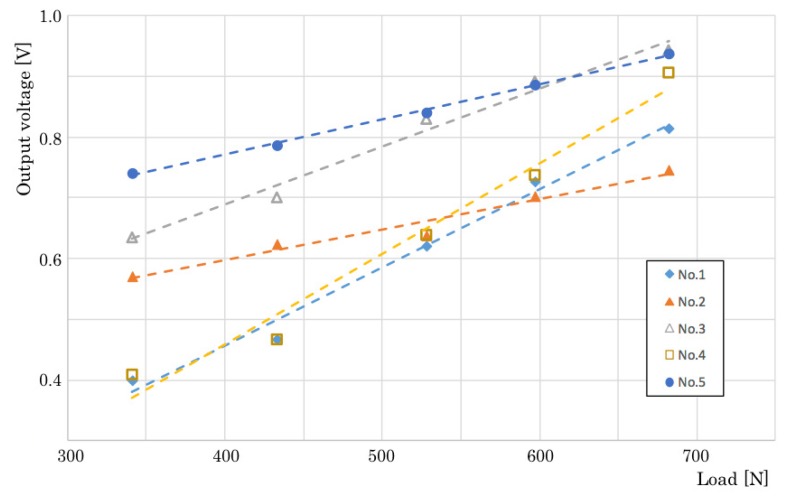
Relation between output voltage and load of sensors.

Figure 5 depicts the output characteristics of five sensors. The vertical and horizontal axes respectively depict output voltage and test loads. The output voltage increases concomitantly with the load until the peak for the maximum load. Subsequently, reverse voltage appears during a slight time as a steady state for removing the load cell from the device. We calculated output characteristics of our prototype sensors using this peak voltage obtained from this test. The results address the linear relation between the sensors and the test load patterns, although the gradients differ among sensors. We consider that the output voltage increases concomitantly with the weight of a person.

**Figure 6:**
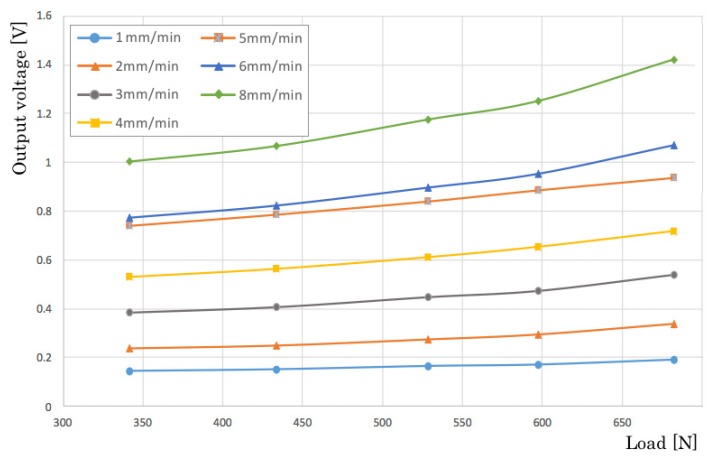
Relation between output voltage and load speed.

**Figure 7:**
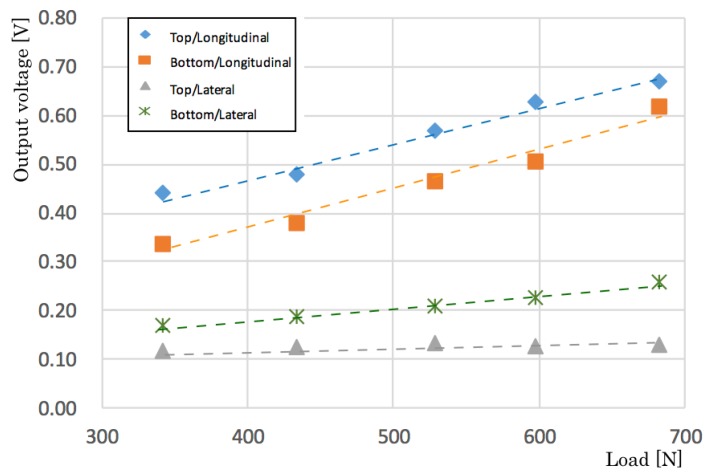
Relation between output voltage and load of side and orientation.

Figure 6 presents results of changing test speeds from 1 mm/min to 8 mm/min step by 1 mm/min. The output voltage increases concomitantly with speed changes that are similar characteristics that resemble the load-test results presented above. Figure 7 depicts characteristics of the side and orientation of the sensors. We evaluated four patterns: a top/longitudinal side, a bottom/longitudinal side, a top/lateral side, and a bottom/lateral side. The top and bottom sides are defined by rivets of the piezoelectric film. The results are depicted in Figure 7. The output voltage of the longitudinal side is 3.12 times higher than that of the lateral side. This directivity characteristic is reflected in the sensor installation to the bed-leaving direction.

### 2.5. Sensor Installation

**Figure 8:**
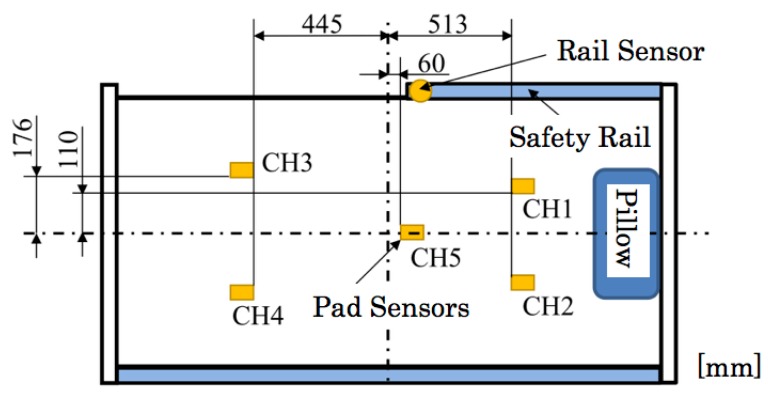
Sensor configuration.

**Figure 9:**
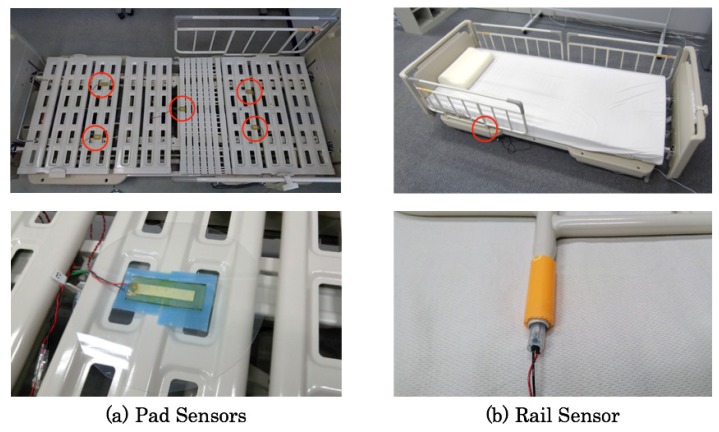
Sensor installation.

**Figure 10:**
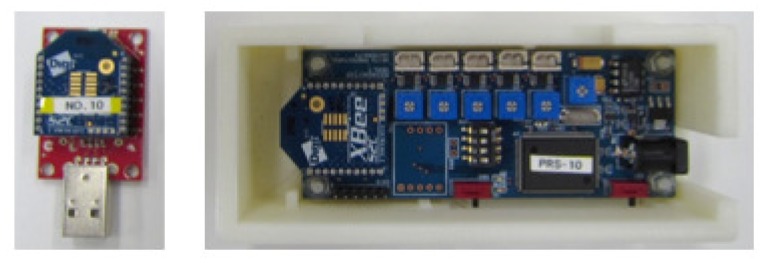
Sensor measurement board with ZigBee module.

To sense the distributed weight of a sleeping body on a bed, pad pressure sensors are installed to five areas between a mattress and a bed frame. Figure 8 depicts the sensor configuration on a bed. The approximate measurement ranges of each sensor are the upper body for channels 1 and 2 (CH1 and CH2), legs for the channels 3 and 4 (CH3 and CH4), and the hip for channel 5 (CH5). A rail sensor is installed to a safety bed rail of one side. Figure 9 depicts photographs of installed sensors. Compared with the pre-installed sensor bed [12], our sensors can be installed on various beds as a post-installation system. Figure 10 depicts a sensor measurement board with a ZigBee module for wireless communication. Sensor signals are transmitted to a monitoring computer via the board. Our system provides easy and simple monitoring using only six sensors of two types and two measurement boards.

## 3. Bed-Leaving Behavior Pattern Recognition Based on Machine-Learning Algorithms

### 3.1. Feature Calculation

Sensor signals are captured with 50 Hz as the default sampling rate for the measurement board, as depicted in Figure 10. Using all features calculated from all obtained sensor signals gives rise to a significant increase of calculation costs. For reducing the total data size, sensor signals are downsampled to 10 Hz. Moreover, signal changes are calculated at 1 s intervals for enhancing features. Let D(t) be the summation of signal changes at time *t*. The absolute difference $\Delta y_{t}$ of momental output values is calculated between t−1 and *t*. For summarizing Δy during 1 s as n=10 in 10 Hz, D(t) are calculated as shown below.

(1) $$D\left(t\right)=\sum_{k=1}^{n}\Delta y_{k}.$$

**Figure 11:**
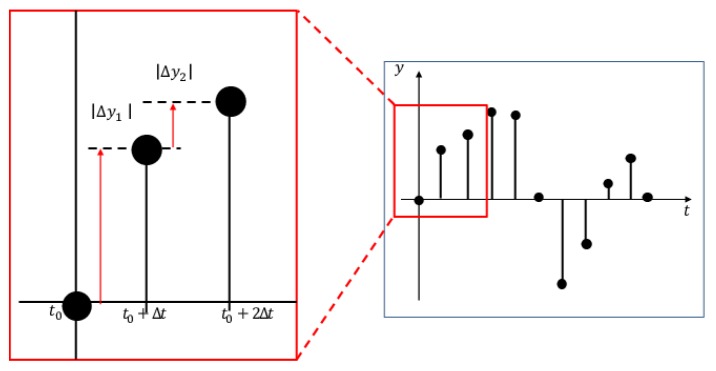
Calculation of signal features.

Figure 11 depicts the outline procedure of feature calculation. Herein, the reason that we used the summation of signal changes for 1 s is because of the output property of piezoelectric elements. Actually, piezoelectric elements have no output voltage with no input force. Output voltage occurs if a dynamic load is attended. After bending, the output voltage returns to 0 V again as a stationary load. For pattern recognition based on machine learning, the status duration with sufficient intervals is desirable for suitable features as correct recognition. Therefore, we infer that false recognition is reduced by the summation of signal changes.

Subsequently, we normalized features for unifying the scale. Let $X_{i}$, $\bar{X}$, and *s* respectively represent input features, mean features, and standard deviation of features. As normalized $D_{i}$, $Z_{i}$ is calculated as

(2) $$Z_{i}=\frac{X_{i}-\bar{X}}{s}.$$

### 3.2. Recognition Algorithms

The aim of this study is to provide a sensor system without setting body parameters as a subject’s profile in advance. We use machine-learning algorithms as a robust approach to absorbing individual differences. Actually, various machine-learning algorithms are present with the feature of easy implementation. For our earlier study [6], we used counter propagation networks (CPNs) because of the advantages of visualizing input feature topology on a category map. For this study, we compared various machine-learning algorithms to recognize behavior patterns for developing a reliable sensor system.

As a comparison target, we selected four machine-learning algorithms: a naive Bayes classifier (NB) [13], k-nearest neighbor (kNN) [14], SVMs [15], and random forests (RF) [16]. These algorithms achieve advanced precision recognition with a small data amount. For our earlier study [6], we constructed recognizers for each subject to learn individual differences of bed-leaving behavior patterns. Each recognizer is optimized in each with limited datasets. In contrast, an insufficient data amount against diverse behavior patterns gives rise to dropped recognition accuracy. Therefore, we attempt to construct a recognizer using datasets of all subjects. Herein, we used the scikit-learn machine-learning library [17] for implementation. The following are outlines for the respective algorithms.

#### 3.2.1. NB

Based on Bayesian theory [18], NB employs supervised learning with the assumption that each feature vector is independent. Let *y* and $x_{n}$ respectively represent class labels and feature vectors. Based on Bayesian theory [18], the following probability is derived.

(3) $$P\left(y|x_{n}\right)=\frac{\left(P\left(y\right)P\left(x_{n}|y\right)\right)}{P(x_{n})}$$

Herein, the joint probability of each feature vector, which is assumed independence, is expressed by the product of the respective probabilities.

(4) $$P\left(x_{n}|y\right)=\prod_{i=1}^{n}P\left(x_{i}|y\right)$$

Therefore, $P(y|x_{n})$ is defined as shown below.

(5) $$P\left(y|x_{n}\right)=\frac{P\left(y\right)\prod_{i=1}^{n}P\left(x_{i}|y\right)}{P(x_{n})}\propto P\left(y\right)\prod_{i=1}^{n}P\left(x_{i}|y\right)$$

Let $\hat{y}$ be a final estimated class label. $\hat{y}$ is derived from the maximum probability as

(6) $$\hat{y}=\underset{y}{\mathrm{argmax}}=P\left(y\right)\prod_{i=1}^{n}P\left(x_{i}|y\right).$$

#### 3.2.2. kNN

As a simple supervised learning algorithm, kNN initially plots all features in learning signals on a vector space. Subsequently, *k* sets of input signals are acquired along with the order of near distance from unknown signal sets. Finally, class labels of unknown signal sets are estimated using a majority voting strategy. Herein, Euclidean distance is used instead of Manhattan distance.

#### 3.2.3. SVM

As a classifier based on supervised learning, SVMs extract a boundary where a margin between two classes has the maximum distance from input signals. A set of distributed signals that is impossible for linear separation is mapped to a high dimensional space using a kernel function for actualizing linear separation. Multi-class features are classified using multiple SVMs with basic two-class classification. For this study, we used two SVM kernel types: linear SVMs (LSVMs) and radial basis function SVMs (RBF-SVMs).

#### 3.2.4. RF

For improving generalization capability, RF comprises ensemble learning algorithms. Initially, weak classifiers are generated from decision trees. Subsequently, decision trees estimate a class label through tracing conditional branches in order from a parent node. Finally, class labels are decided with majority voting from results estimated from respective decision trees.

#### 3.2.5. CPN

CPNs are supervised neural networks that extend from self-organizing maps (SOMs) [19] as unsupervised neural networks. Data topologies are preserved with the competition and neighborhood learning strategy. The CPN network architecture comprises three layers: an input layer, a mapping layer, and a Grossberg layer.

These weights are initialized randomly. Subsequently, a unit on the mapping layer that minimizes the distance calculated from the Euclidean distance of input data $x_{i}$ and $w_{i,p,q}$ is sought. This unit is defined as *c*. The weights $w_{i,p,q}$ of neighbor units inside of *c* are updated. Moreover, $w_{p,q,j}$ are updated using teaching signals $T_{j}$ at time *t*.

Let $w_{r,s}\left(t\right)$ and $w_{s,k}\left(t\right)$ respectively denote weights between input layer *r*(1≤r≤R) and Kohonen layer unit *s*(1≤s≤S) and weights between Grossberg layer *k*(1≤k≤K) to Kohonen layer unit *s* at time *t*. Before learning, $w_{r,s}\left(t\right)$ are initialized randomly. Using the Euclidean distance between $y_{r}\left(t\right)$ and $w_{r,s}\left(t\right)$, a winner unit $c_{s}\left(t\right)$ is sought for the following.

(7) $$c_{s}\left(t\right)=\underset{1\leq s\leq S}{\mathrm{argmin}}\sqrt{\sum_{r=1}^{R}{\left(y_{r}\left(t\right)-u_{r,s}\left(t\right)\right)}^{2}}.$$

A neighborhood region $\psi_{cpn}\left(t\right)$ is set from the center of $c_{s}$ as the following.

(8) $$\psi_{cpn}\left(t\right)=\left\lfloor \psi_{cpn}\left(0\right)\cdot S\cdot \left(1-\frac{t}{Z_{cpn}}\right)+0.5\right\rfloor ,$$

where $Z_{cpn}$ stands for the maximum learning iteration. Subsequently, $w_{r,s}$ and $w_{s,k}$ in $\psi_{cpn}\left(t\right)$ is updated as shown below.

(9) $$w_{r,s}\left(t+1\right)=w_{r,s}\left(t\right)+\beta \left(t\right)\left(y_{r}\left(t\right)-w_{n,m}\left(t\right)\right),$$

(10) $$w_{s,k}\left(t+1\right)=w_{s,k}\left(t\right)+\gamma \left(t\right)\left(z_{l}\left(t\right)-w_{n,m}^{j}\left(t\right)\right),$$

where β(t) and γ(t) are learning coefficients that decrease along with learning progress.

This process is repeated until the maximum number of learning iterations is reached. Finally, unit labels $L_{j}$ are decided as a result of maximized $w_{s,k}$ against the unit *k* on Grossberg layer. After learning, CPNs provide a recognition result based on winner-take-all competition for a set of input signals.

## 4. Datasets

### 4.1. Target Behavior Patterns

**Figure 12:**
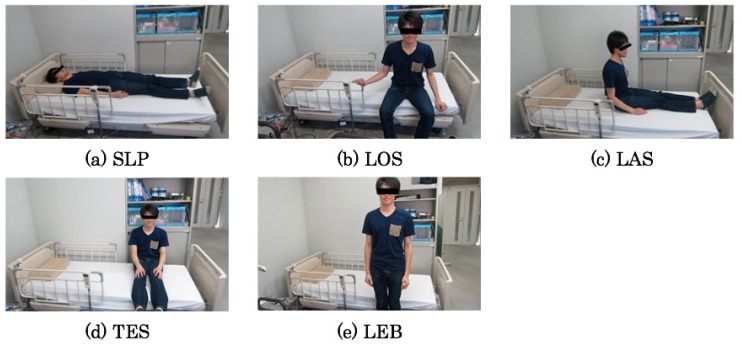
Target behavior patterns.

Figure 12 depicts photographs in each pose for the target behavior patterns. The following are features and estimated sensor responses for the respective patterns.

**SLP**  Sleeping: a subject is sleeping on a bed normally.
**LOS**  Longitudinal sitting: a subject is sitting longitudinally on a bed after rising.
**LAS**  Lateral sitting: a subject is sitting laterally on a bed after turning the body from longitudinal sitting.
**TES**  Terminal sitting: a subject is sitting in the terminal position on a bed trying to leave a bed. Rapid and correct detection is necessary because of the terminal situation for leaving a bed.
**LEB**  Left a bed: a subject has left the bed. Herein, sensor responses disappear in the status of losing consciousness or a life crisis. For such circumstances, monitoring devices such as electrocardiographs are used. Such circumstances are beyond our prediction targets.

### 4.2. Datasets Obtained for Conditions

We obtained bed-leaving behavior pattern datasets at a simulated experimental room that resembled a clinical site. We used an electro-actuation bed (KA-36121R; Paramount Bed Co., Ltd.) equipped with three actuators for reclining the back and feet panels and for adjusting its height. We obtained datasets without using the back plate reclining function for avoiding load pattern changes on the bed. The route for a subject to leave from the bed is restricted to one side with two attached safety rails.

The subjects were 10 persons: nine men and one woman. Table 1 presents profiles of all subjects. We set two protocols to obtain different characteristic datasets. The first protocol comprises the same procedures as those of our earlier study [6]. Each subject switched their behavior patterns of five types with 20 s intervals. For this study, we call them continuous datasets (CDS). We obtained 10 sets of CDS from each subject. Herein, the data sampling rate was set to 50 Hz.

The second protocol comprises behavior patterns as discontinuous datasets. The order and duration of bed-leaving behavior patterns are various along with body parameters and health conditions in each subject. For example, behavior patterns from LOS to SLP without changing to LAS occurred frequently. We consider that the employment of our sensor system at a clinical site gives rise to dramatically lower recognition accuracy. As a basic consideration for aiming a practical application, we obtained datasets without fixed sequences or time intervals for subjects of their basic behavior pattern transitions. We designate them as discontinuous datasets (DDS). The data acquisition period was set to 15 min per person.

For calculating recognition accuracy, ground truth (GT) labels are indispensable to DDS. However, the burden to allocate GT labels is excessively high because each subject moved freely for 15 min. Therefore, we used a depth camera to record video images for annotation. We allocated GT labels to DDS manually from video image observation.

### 4.3. Sensor Output Signals

**Figure 13:**
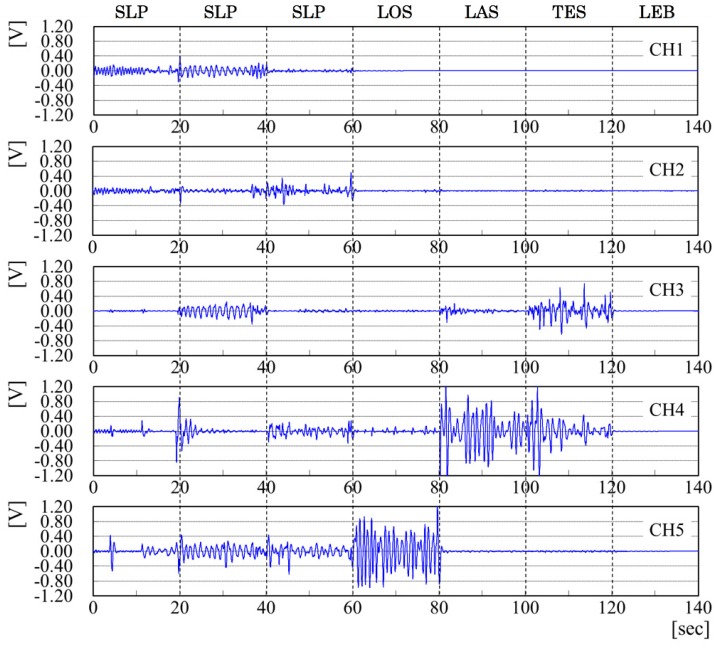
Output signals from pad sensors.

Figure 13 depicts output signals from the pad sensors. The vertical and horizontal axes respectively represent the output voltage and translation time in seconds. The voltage range is up to ±1.2 V. Along with time transitions, each subject changes their behavior patterns in the order as depicted in Figure 12. During 60 s from the initial point, a subject was sleeping on the bed with turning of the body. The output signals in respective channels were changed slightly.

The output signals from CH5, which correspond to the bed center, have come to be high in LOS. This tendency demonstrates that the upper body weight was concentrated to this channel. The output signals from CH4 are salient in LAS because of turning the body to the lateral bed direction for the transition to LEB. Salient output signals in TES are changed from CH4 to CH3. The output signals in LEB are disappeared completely.

**Figure 14:**
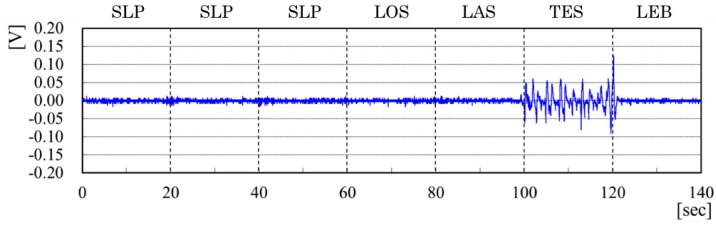
Output signals from rail sensor.

Figure 14 depicts output signals from the rail sensor. No salient signals are presented in SLP, LOS, and LAS. The output signals are noticeable in TES. After the boundary between TES and LEB, no output signals are presented. The output signal tendency from the rail sensor indicates a selective feature in TES compared with those of other behavior patterns.

## 5. Evaluation Experiment

### 5.1. Evaluation Criteria

Let $T_{num}$ and $G_{num}$ respectively be the total numbers of test signals and GT labels. For evaluation criteria, the recognition accuracy *R* for a test dataset is defined as

(11) $$R=\frac{T_{num}}{G_{num}}\times 100\;\left[\%\right].$$

Herein, we define mean *R* as $R_{mean}$. Moreover, we define *R* of SLP, LOS, LAS, TES, and LEB as $R_{SLP}$, $R_{LOS}$, $R_{LAS}$, $R_{TES}$, and $R_{LEB}$.

We used *K*-Fold cross-validation for evaluating results along with machine-learning and evolutional-learning approaches. Herein, we set K=5 based on the results of earlier studies [20,21].

We conducted four evaluation experiments using behavior pattern datasets of two types. Table 2 summarizes experimental details.

### 5.2. Comparison Results of Learning Algorithms

We evaluated recognition accuracy of machine-learning algorithms using CDS. Table 3 depicts comparison results. We used CPNs as a discriminator for our earlier study [6]. As a comparison result, $R_{mean}$ using CPNs was 75.4%, which is the second lowest of six algorithms. In contrast, $R_{mean}$ using RF was 91.1% that the highest. For all behavior patterns except of LEB, $R_{mean}$ using RF were higher than those of other algorithms. For LEB, LSVM provided the highest recognition accuracy.

As a commonplace tendency for all algorithms, $R_{SLP}$ achieved the highest. In contrast, $R_{LOS}$, $R_{LAS}$, and $R_{TES}$ are smaller than 90.0%. We consider that the recognition accuracies for these three behavior patterns must be high because our system intends to predict bed-leaving behavior. Therefore, we examine measures to improve recognition accuracies for these three behavior patterns from the viewpoint of datasets and discriminators.

### 5.3. Experimental Results for CDS

**Figure 15:**
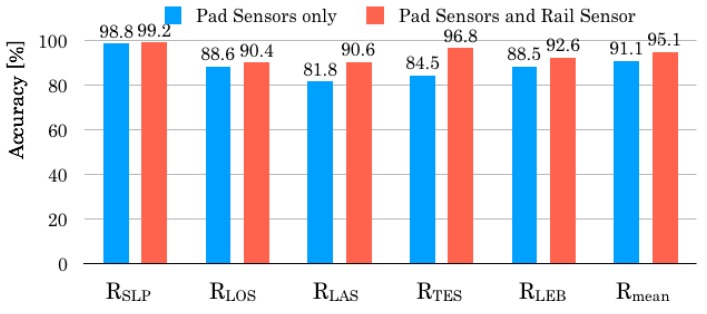
Comparison results of recognition accuracies.

Using CDS, we evaluated the capabilities of our originally developed sensors of two types. Figure 15 depicts comparison results of $R_{mean}$ for the solely used pad sensors and the combined sensors with pad sensors and a rail sensor. Comparison of the results shows that $R_{mean}$ of the combined sensors is 4.0% higher than that of the pad sensors. Recognition accuracies in respective behavior patterns are improved from 0.4% up to 12.3%. Particularly, $R_{TES}$ exhibits the maximum improvement. Moreover, the experiment result demonstrates that the rail sensor, which detects the grasp of a safety rail in TES intensively, contributes to the improvement of the overall recognition accuracy.

As shown in Figure 14, the output voltage from the piezoelectric film in the rail sensor occasionally exceeded 0.1 V when a subject was in TES. We infer that sufficient recognition accuracy is obtainable using the rail sensor solely if we set the target-only TES. However, we consider that false recognition occurred between TES and LEB, especially for the immediate transition from TES to LEB. Although sensor signals should be approximately 0 V in LEB, sharp signals are outputted. This tendency occurs when a subject tries to leave from the bed with holding or shaking of a safety rail, which enhances vibration. Therefore, we infer that the combination between the rail sensor and the pad sensors is the best for a practical use because of avoiding the problem that is occurred in the case of a solely used rail sensor. Moreover, false recognition to LEB is avoidable accurately because the recognition accuracy of the combined sensors obtained relative superior improvement for LAS, TES, and LEB.

We examine detailed recognition results obtained using a confusion matrix. Table 4 and Table 5 respectively present confusion matrixes for the pad sensors and the combined sensors. Specifically examining TES with the highest recognition accuracy, the number of false recognition instances was reduced in all behavior patterns after appending the rail sensor. Particularly, the number of false recognitions to LEB was reduced from 30 signals to 4 signals. We infer that the correct discrimination between TES and LEB engenders improved recognition accuracy.

Although the use of the rail sensor was aimed at improved $R_{TES}$, $R_{LAS}$ was improved from 81.8%–90.6% as a subsidiary contribution. The number of false recognition instances was reduced, except for SLP. Particularly, the number of false recognition instances for TES was decreased to 26 times. The improved $R_{TES}$ after appending the rail sensor produces decreased false recognition instances of LAS. As a result, correct recognition instances, except for TES, produces an improved $R_{mean}$. We demonstrated that the addition of a sensor that can reliably recognize a single posture engendering improvement of $R_{mean}$. The combined sensors have clear benefits for bed-leaving behavior recognition when compared to other configurations.

### 5.4. Experiment Results for DDS

Table 6 denotes $R_{mean}$ for each subject for DDS. Compared with that of CDS as depicted in Figure 15, recognition accuracies were lower in all five behavior patterns. Especially, $R_{LAS}$ was drastically lower. This experimentally obtained result revealed that recognition accuracy, except for $R_{SLP}$, which was the highest accuracy among five behavior patterns, was strongly affected by randomness in DDS.

The disparity of detailed recognition accuracies in respective subjects was from 43.3% as the lowest to 95.3% as the highest. Compared with the mean accuracy of 75.2%, the recognition accuracies were above for seven subjects and below for the remaining three subjects. Therefore, significant lower accuracy for specific subjects decreased the overall recognition accuracy. We infer that this tendency is influenced by individual differences in behavior patterns. Each subject played predetermined behavior patterns in CDS and free behavior patterns in DDS. We consider that the recognition accuracy was significantly lower because behavior patterns were various among the subjects in DDS.

Table 7 depicts the confusion matrix for all subjects. Table 8 presents the confusion matrix for Subject C, with the lowest recognition accuracy. Numerous signals were falsely recognized to SLP. Particularly, correct recognition in LEB was merely 2 of 179 signals. Other signals were falsely recognized to SLP. This trend demonstrated that false recognition occurred in the state that SLP and other behavior patterns were not distinguished. In DDS, $R_{mean}$ was dramatically lower in particular subjects. We consider that this is attributable to behavior variations of subjects between learning datasets and test datasets. Therefore, we consider that $R_{mean}$ improves if learning datasets contain diversity.

### 5.5. Integration of Learning Datasets

For applying our system at a clinical site, learning dataset preparation for each subject might be troublesome and time-consuming. In addition, preserving accuracy has come to be a problem of system reliability because generalization was dropped in DDS. Therefore, we attempt to construct a generic classifier combined with learning datasets for all subjects as depicted in Table 1. For this experiment, we used CDS for learning and DDS for validation.

Table 9 depicts recognition accuracies obtained before and after the integration of learning datasets. Recognition accuracies in six of ten subjects were improved. Particularly, recognition accuracies of Subjects C and G were improved notably. Although recognition accuracies of four subjects dropped, three of them remained dropped percentages up to 2.0%. However, with regard to Subject E, $R_{mean}$ decreased 5.6 percentage points, especially for steep drops in $R_{LOS}$ and $R_{LAS}$.

**Figure 16:**
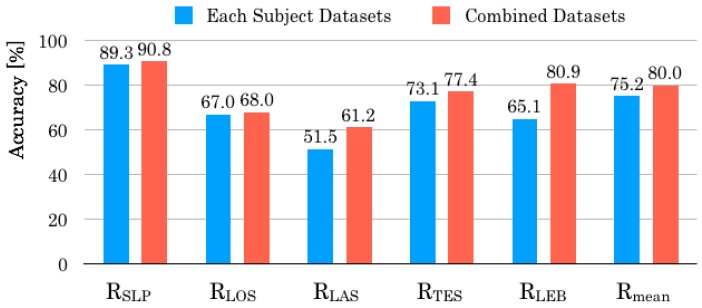
Comparison result of recognition accuracy in each behavior pattern.

Figure 16 depicts the comparison result of recognition accuracy for each behavior pattern. All recognition accuracies were improved in the integrated datasets. Particularly, $R_{LEB}$ improved 15.8 percentage points. Table 10 denotes the confusion matrix for all subjects after the integration of validation datasets. Compared with the results depicted in Table 7, the numbers of false recognition instances were lower in all behavior patterns. Although the false recognition instances for SLP were numerous in Table 7, these results were improved in Table 10. Moreover, false recognition instances appeared frequently in behavior patterns that were close to GT labels except of LAS. We consider that it is a challenging task to recognize intermediate states of two neighbor behavior patterns which change their body among respective behavior patterns. To reduce false recognition instances, we infer that it is necessary to use a method to maintain the previous status until the recognition becomes stable if a present recognition result differs from the previous one. In contrast, false recognition instances of LAS were divided to the other four behavior patterns evenly. We infer that this tendency makes it difficult to distinguish LAS from other behavior patterns.

For the integration of learning datasets among subjects, we achieved not only maintenance of generalization performance for DDS, but also prevention of false recognition extremely. Although recognition accuracy was improved overall, several subjects showed low recognition accuracy. We conclude that this learning strategy does not ensure whole improve recognition accuracy. For improving this system, we consider that important prerequisites remain as the following: to improve generalization capability for collecting numerous datasets from numerous subjects, to change learning datasets along with subject profiles in terms of height or weight, to perform incremental learning without stopping the system until sufficient accuracy is obtained, and to construct learning datasets specialized to each subject temporally.

### 5.6. Discussion

For this study, we developed the sensor system that is inexpensive, convenient, and maintainable with advanced QoL for care recipients. Actually, as described in the introduction, using a camera as a bed monitoring sensor can provide a low-cost system that can obtain much information from subjects. However, it is still a challenging task to predict behavior patterns obtained from images, even when state-of-the-art computer vision technologies are used. For example, as a deep-learning-based approach, OpenPose [22] does not handle sleeping or laying positions. Therefore, medical staff members must observe images directly. Moreover, we have to consider aspects of human rights and QOL, especially, it is impossible not only to monitor numerous subjects simultaneously with a few operators but also to recognize behavior patterns related to bed-leaving using only sensor responses, even when detailed analyses are conducted, because behavior patterns differ among people. Furthermore, monitoring using a camera imposes a mental load on patients because they feel as though they are under surveillance all daytime and nighttime. However, we have not evaluated this sensor system at hospitals or care facilities. We would like to subjective and objective evaluation to validate our sensor system in a clinical environment without the use of cameras.

## 6. Conclusions

This paper presented the bed-leaving behavior recognition system that comprises pad sensors installed on a bed, a rail sensor inserted in a safety rail, and a behavior pattern recognizer based on machine learning algorithms. We obtained benchmark datasets of continuous and discontinuous behavior patterns from 10 subjects. The experimentally obtained results revealed that RF obtained the highest recognition accuracy in our benchmark datasets. Compared with our earlier study, results obtained using CPNs, the recognition accuracies were improved by 20.7% for LOS and 21.9% for TES. After appending the rail sensor to the pad sensors, the mean recognition accuracy improved 4.0 percentage points, including a 12.3 percentage point improvement for TES. Regarding the difference in behavior pattern transitions, the mean recognition accuracy decreased 22.9 percentage points in discontinuous datasets. For improving the generalization of our system, datasets of all subjects were combined for learning. The mean recognition accuracy improved 4.8 percentage points, especially improved considerably for two subjects.

For our future work, we aim to apply our proposed sensor system to a clinical site such as care facilities or single senior’s homes for security and safety observation that simultaneously maintains QOL and privacy. We will achieve steady detection to expand the application range of our method and thereby increase the number of subjects. Additionally, we must demonstrate the system reliability for conducting long-term monitoring.

## Figures and Tables

**Table 1:** Profile of subjects.

Subject	A	B	C	D	E	F	G	H	I	J
Height [cm]	161	169	177	168	170	167	177	178	170	165
Weight [kg]	51	66	91	51	60	61	84	80	78	70
Sex	F	M	M	M	M	M	M	M	M	M

**Table 2:** Experimental conditions.

Section	Learning Dataset	Test Dataset	Discriminator
Section 5.2	CDS	CDS	Each Subject
Section 5.3	CDS	CDS	Each Subject
Section 5.4	CDS	DDS	Each Subject
Section 5.5	CDS	DDS	All Subject

**Table 3:** Comparison results of learning algorithms [%].

Algorithm	$R_{\mathrm{SLP}}$	$R_{\mathrm{LOS}}$	$R_{\mathrm{LAS}}$	$R_{\mathrm{TES}}$	$R_{\mathrm{LEB}}$	$R_{\mathrm{mean}}$
NB	98.7	32.2	27.4	25.8	0.9	53.2
kNN	98.5	85.0	79.5	80.8	86.1	89.0
LSVMs	96.2	63.8	64.4	51.1	89.4	78.2
RBF-SVMs	98.0	62.4	64.6	67.9	88.8	81.0
RF	98.8	88.6	81.8	84.5	88.5	91.1
CPNs	88.1	67.9	59.7	62.5	75.9	75.4

**Table 4:** Confusion matrixes for the results of pad sensors.

	SLP	LOS	LAS	TES	LEB
SLP	1047	5	3	3	2
LOS	21	443	14	3	19
LAS	12	9	319	36	14
TES	3	8	18	321	30
LEB	2	6	12	18	292

**Table 5:** Confusion matrixes for the results of pad sensors and rail sensors.

	SLP	LOS	LAS	TES	LEB
SLP	1052	3	3	0	2
LOS	19	449	14	1	17
LAS	13	3	354	10	10
TES	1	2	8	365	4
LEB	2	10	12	3	303

**Table 6:** Recognition accuracies in each subject for DDS [%].

Subject	$R_{\mathrm{SLP}}$	$R_{\mathrm{LOS}}$	$R_{\mathrm{LAS}}$	$R_{\mathrm{TES}}$	$R_{\mathrm{LEB}}$	$R_{\mathrm{mean}}$
A	67.1	65.8	65.6	69.4	84.5	68.9
B	97.8	51.4	22.8	79.6	78.8	82.4
C	95.2	66.0	8.8	10.9	1.1	43.3
D	98.9	84.8	42.2	89.6	94.3	84.6
E	93.9	78.1	60.3	88.1	82.9	83.7
F	99.8	89.2	95.1	90.3	95.2	95.3
G	51.3	43.9	47.0	89.1	15.0	51.6
H	98.1	64.0	51.1	78.3	79.7	73.5
I	95.0	71.0	57.7	61.7	88.9	86.5
J	95.9	55.7	64.6	74.0	30.3	82.1
Average	89.3	67.0	51.5	73.1	65.1	75.2

**Table 7:** Confusion matrix of all subjects.

	SLP	LOS	LAS	TES	LEB
SLB	**4097**	356	142	5	26
LOS	312	**1283**	162	11	96
LAS	179	201	**795**	116	164
TES	295	21	72	**974**	135
LEB	183	55	26	58	**641**

**Table 8:** Confusion matrix of Subject C.

	SLP	LOS	LAS	TES	LEB
SLP	**335**	17	0	0	0
LOS	52	**171**	1	1	34
LAS	141	4	**16**	9	11
TES	286	0	0	**35**	0
LEB	177	0	0	0	**2**

**Table 9:** Recognition accuracies before and after integration of learning datasets [%].

Subject	Before	After	Difference
A	68.9	67.1	−1.8
B	82.4	86.9	4.5
C	43.3	77.9	34.6
D	84.6	84.7	0.1
E	83.7	78.1	−5.6
F	95.3	93.5	−1.8
G	51.6	66.4	14.8
H	73.5	77.4	3.9
I	86.5	87.0	0.5
J	82.1	80.8	−1.3
Average	75.2	80.0	4.8

**Table 10:** Confusion matrix.

	SLP	LOS	LAS	TES	LEB
SLP	**4190**	310	53	6	67
LOS	315	**1284**	162	13	90
LAS	150	131	**909**	117	148
TES	48	45	141	**1166**	97
LEB	21	30	37	74	**801**

## Abbreviations

The following abbreviations are used in this manuscript:

CDS	Continuous DataSets
CPNs	Counter Propagation Networks
DDS	Discontinuous DataSets
GT	Ground Truth
IR	InfRared
kNN	k-Nearest Neighbor
LAS	LAteral Sitting
LEB	LEft a Bed
LOS	LOngitudinal Sitting
LSVMs	Linear Support Vector Machines
NB	Naive Bayes
PET	Polyethylene Terephthalate
QoL	Quality of Life
RBF-SVMs	Radial Basis Function Support Vector Machines
SOMs	Self-Organizing Maps
SLP	SLeePing
SVMs	Support Vector Machines
TES	TErminal Sitting
RF	Random Forests
